# Influence of Cervical Muscle Fatigue on Musculo-Tendinous Stiffness of the Head-Neck Segment during Cervical Flexion

**DOI:** 10.1371/journal.pone.0139333

**Published:** 2015-09-29

**Authors:** Raphaël Portero, Franck Quaine, Violaine Cahouet, Marc Léouffre, Christine Servière, Pierre Portero

**Affiliations:** 1 Département d’Automatique, GIPSA Lab, UMR CNRS 5216, Université Joseph Fourier, Grenoble, France; 2 Bioingénierie, Tissus et Neuroplasticité, EA 7377, Université Paris-Est, UPEC, Créteil, France; 3 Service de Rééducation Neuro-Orthopédique, Hôpital Rothschild (AP—HP), Paris, France; University of Rome Foro Italico, ITALY

## Abstract

**Aim:**

The aim of this study is to determine if the fatigue of cervical muscles has a significant influence on the head-neck segment musculo-tendinous stiffness.

**Methods:**

Ten men (aged 21.2 ± 1.9 years) performed four quick-release trials of flexion at 30 and 50% MVC before and after the induction of muscular fatigue on cervical flexors. Electromyographic activity was recorded on the sternocleidomastoids (SCM) and spinal erectors (SE), bilaterally. Musculo-tendinous stiffness was calculated through the quick-release method adapted to the head-neck segment.

**Results:**

We noticed a significant linear increase of the head-neck segment musculo-tendinous stiffness with the increase of exertion level both before (P < 0.0001) and after the fatigue procedure (P < 0.0001). However, this linear relationship was not different before and after the fatigue procedure. EMG analysis revealed a significant increase of the root mean square for the right SCM (P = 0.0002), the left SCM (P < 0.0001), the right SE (P < 0.0001), and the left SE (P < 0.0001) and a significant decrease of the median power frequency only for the right (P = 0.0006) and the left (P = 0.0003) SCM with muscular fatigue.

**Discussion:**

We did not find significant changes in the head-neck segment musculo-tendinous stiffness with fatigue of cervical muscles. We found a significant increase in EMG activity in the SCM and the SE after the induction of fatigue of the SCM. Our findings suggest that with fatigue of cervical flexors, neck muscle activity is modulated in order to maintain the musculo-tendinous stiffness at a steady state.

## Introduction

Head stabilization plays a major role in proprioception, collection of sensory information and protection of the spinal cord. Head stabilization depends on multiple factors such as neck muscle strength [1], the reflex response pattern to external perturbations [2,3], and global mechanical properties of the neck [4]. Head stabilization is thus strongly linked to head-neck musculo-tendinous stiffness. The musculo-tendinous stiffness is the stiffness of the series elastic component (SEC) of a muscle or a group of muscles. The function of SEC is to transmit the strength generated by the contractile component to the corporal segments. The SEC is located in tendon, connective tissue, and actin-myosin cross-bridge interaction [5]. In *in vivo* conditions, the musculo-tendinous stiffness at a joint is the resultant stiffness of all muscular groups crossing the joint and may involve neuromuscular patterns between agonist and antagonist muscles to maintain the mechanical balance as suggested for the lumbar spine [6,7].

The quick-release (QR) method popularized by Goubel and Pertuzon (1973) [8] can be used to evaluate SEC stiffness. It has been shown that during the initial isometric contraction at a constant exertion level, the elastic energy is stored in the SEC. When the system is suddenly released, this energy is instantaneously restored and changes in SEC strength and length are analyzed before the occurrence of reflex activity in order to obtain the SEC stiffness [8]. Recently, this method was successfully adapted to the neck muscles and the authors have shown a significant increase in the musculo-tendinous stiffness with increasing torque [9].

Muscular fatigue is defined as an inability to maintain force or power output during sustained or repetitive contractions [10]. It is attributed to many different mechanisms, ranging from the accumulation of metabolites within muscle fibers (peripheral factors) to the generation of an inadequate motor command in the motor cortex (central factors) [11]. Thus, with fatigue it is necessary to recruit a greater number of motor units to maintain a pre-determined exertion level [12]. However, no agreement has been reached that muscle fatigue modifies musculo-tendinous stiffness. Indeed, contradictory relationships are reported. For example, it has been noticed that the musculo-tendinous stiffness of the elbow flexors and extensors obtained with QR perturbations [13] or oscillation perturbations [14] decreases with fatigue. Concordant insights were observed on isolated fibers from frog muscles through a sinusoidal length oscillation protocol [15,16]. Conversely, it has been shown that muscular fatigue induced by submaximal contractions during stretch-shortening cycles was associated with a significant increase of the musculo-tendinous stiffness on isolated rat muscle [17]. This was attributed to the enhancement of the number of attached cross-bridges during fatigue [17,18].

It is well documented that neuromuscular control factors contributing to spinal stability are influenced by fatigue of trunk muscles. Fatigue alters muscle reflex activity [19–22] and modifies muscle recruitment leading to an increase in coactivation to modulate spinal global stiffness [23–25]. At the cervical spine level, the effects of neck muscle fatigue have been evaluated during physical activities such as the scrum in rugby [26], aerial combat [27,28], and also through motor tasks specifically involving cervical muscles [29]. It has been shown that muscular fatigue has deleterious effects on head stabilization [27,28] and on neck muscle strength [27] but there are no data about changes in musculo-tendinous stiffness.

Thus, the question of whether the head-neck musculo-tendinous stiffness changes with fatigue is of great interest and remains unexplored. The aim of this study was to investigate the effects of cervical flexor muscle fatigue on the head-neck musculo-tendinous stiffness through the application of QRs.

## Materials and Methods

### Subjects

Ten healthy men (21.2 ± 1.9 years) with no history of neck and / or back pain volunteered to participate in the study. Each subject gave written informed consent before participating. The experimental design was approved by the Ethical Committee of Joseph Fourier University and was conducted in accordance with the Declaration of Helsinki (last modified in 2004).

### Experimental device

**Fig 1:**
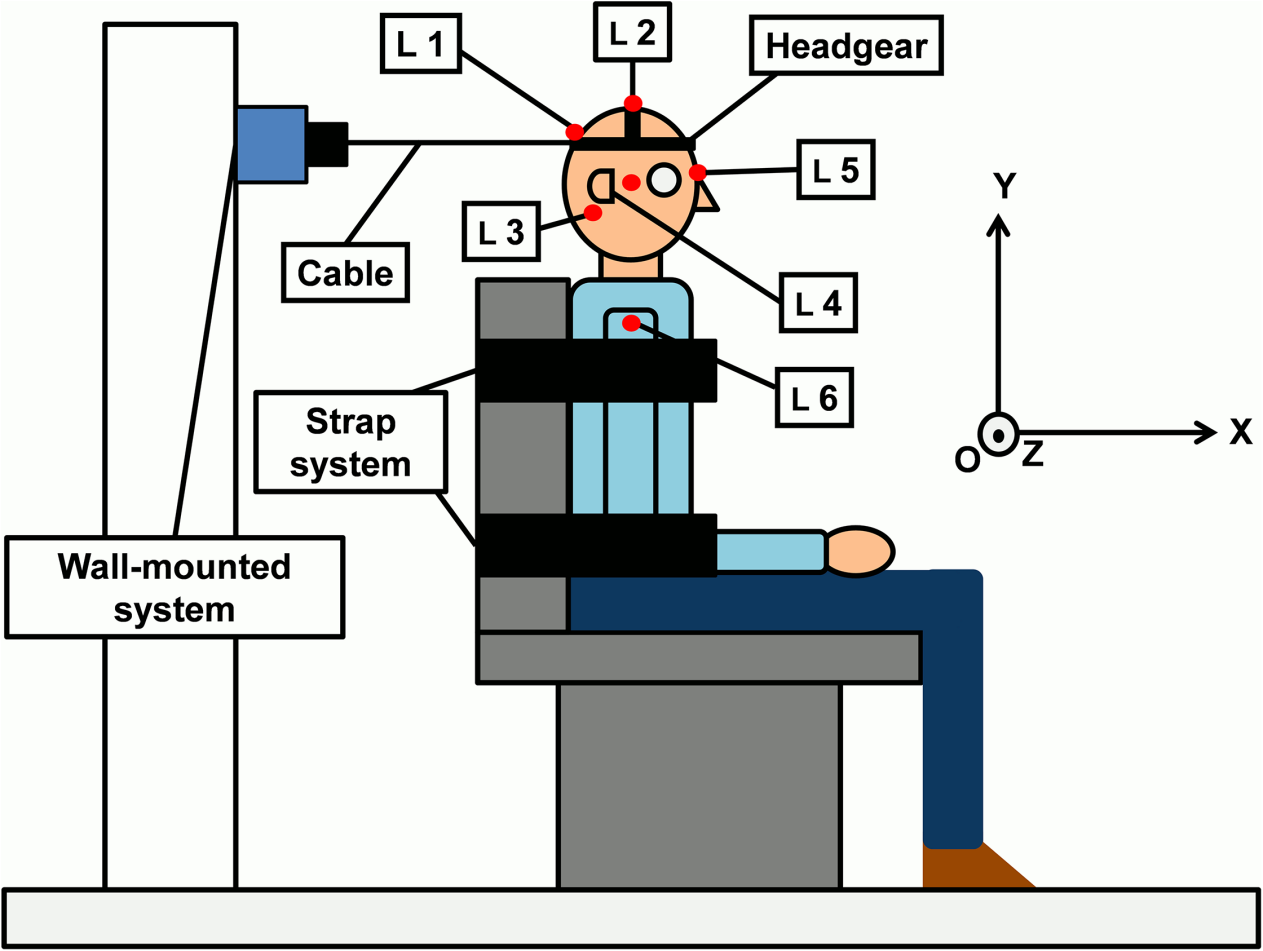
Subject placed in sitting. The headgear is positioned on the head and fixed with the cable to the wall-mounted system composed of the electromagnet and the load cell. Kinematics of the five LEDs positioned on the head (L 1, L2, L3, L 4, and L 5) were measured with respect to the LED 6 (L 6), placed on the shoulder.

Subjects were seated on an adjustable seat with their head in a constrained specific upright posture with the Frankfurt plane parallel to the ground and defined as the neutral position (Fig 1). A two-point strap system restrained the upper and lower trunk to the backrest of the seat to isolate the head-neck segment dynamics. A headgear was positioned with the head in the neutral position. A cable attached to the headgear was linked to a wall-mounted system composed of a 1-D load cell (Eaton^®^ (Cleveland, USA), model Lebow 3167, capacity: 500 N) coupled to an electromagnet (Mecalectro^®^ (Massy, France), model P.5.18.44, maximum gripping force: 500 N). The line of pull of the cable was horizontal. Deactivation of the electromagnet triggered the release of the cable. Force intensity (F) from the force sensor was presented on a monitor positioned at eye-level in front of the subject to indicate the target force value.

The EMG activity of the left and right sternocleidomastoid (SCM_L_ and SCM_R_, respectively) and left and right spinal erector (SE_L_ and SE_R_, respectively) muscles was recorded. Two surface electrodes per muscle (Biopac Systems Inc. (Santa Barbara, USA), model EL503) were used. They were positioned according to the SENIAM (Surface Electromyography for Non-Invasive Assessment of Muscle, SENIAM 5, 1997) project recommendations. The electrodes were positioned during a small isometric contraction of the cervical muscles with the head-neck segment in the neutral position. A common ground electrode was positioned on a bony site (the clavicle).

Three-dimensional displacements of 6 light-emitting diodes (LEDs) were measured using the Optotrak Motion Capture System (Northern Digital Inc. (Waterloo, Canada)). Displacements were expressed in the global reference system $\left(\text{O},\vec{\text{X}},\vec{\text{Y}},\vec{\text{Z}}\right)$ (Fig 1). Diodes were positioned on the subjects as proposed by Portero et al. (2013) [9] (LED 1: junction between cable and headgear, on the line of action of the isometric strength developed by the head-neck segment; LED 2: vertex; LED 3: mastoid process; LED 4: temporal bone, just above the zygomatic arch; LED 5: glabella; LED 6: acromion process) (Fig 1).

Strength and EMG data were collected at 2000 Hz (anti-aliasing filter at 500 Hz), amplified, and recorded by a Biopac acquisition board (Biopac Systems Inc. (Santa Barbara, USA), model EMG100C). The EMG signals were filtered with a band-pass filter (30–400 Hz). Displacement signals of the LEDs were sampled at 200 Hz, processed with respect to the shoulder reference point LED 6, and low-pass filtered with a Butterworth reverse-forward filter (cut-off frequency: 20 Hz; fourth order).

### Experimental procedure

The experimental procedure consisted of a warm-up session followed by the maximal voluntary contraction evaluation and QRs for non-fatigued muscles. The subjects then began the fatigue procedure and ended the experiment with QRs for fatigued muscles.

Once positioned, the subjects performed 15 min of warm-up neck flexions at their own intensity. After the warm up, the subjects performed two maximal voluntary isometric neck flexions sustained for 5 sec. The maximal peak force was considered the MVC value. Once the MVC was determined, subjects were instructed to pull on the cable at 30% or 50% MVC. When the subject reached the force plateau, the experimenter deactivated the electromagnet without the subject knowing. Two QRs were performed at 30% MVC and two at 50% MVC target force intensities in a random order. The fatigue procedure then followed. It consisted of intermittent exercises at moderate intensity interspersed with rest periods. The subjects were asked to sustain five isometric contractions in the neutral position for 30 s in flexion at 50% MVC separated by rest periods of 30 s. This procedure inspired by Schieppati et al. (2003) [22] was chosen after pre-tests to ensure that the subjects were able to perform the experiment until the end. After the fifth fatigue contraction, the subjects re-did the same QRs procedure for fatigued muscles resulting in four tests (two QRs at 30% MVC and two at 50% MVC in a random order).

No subject reported any discomfort following the QR.

### Calculated parameters

#### Musculo-tendinous stiffness

**Fig 2:**
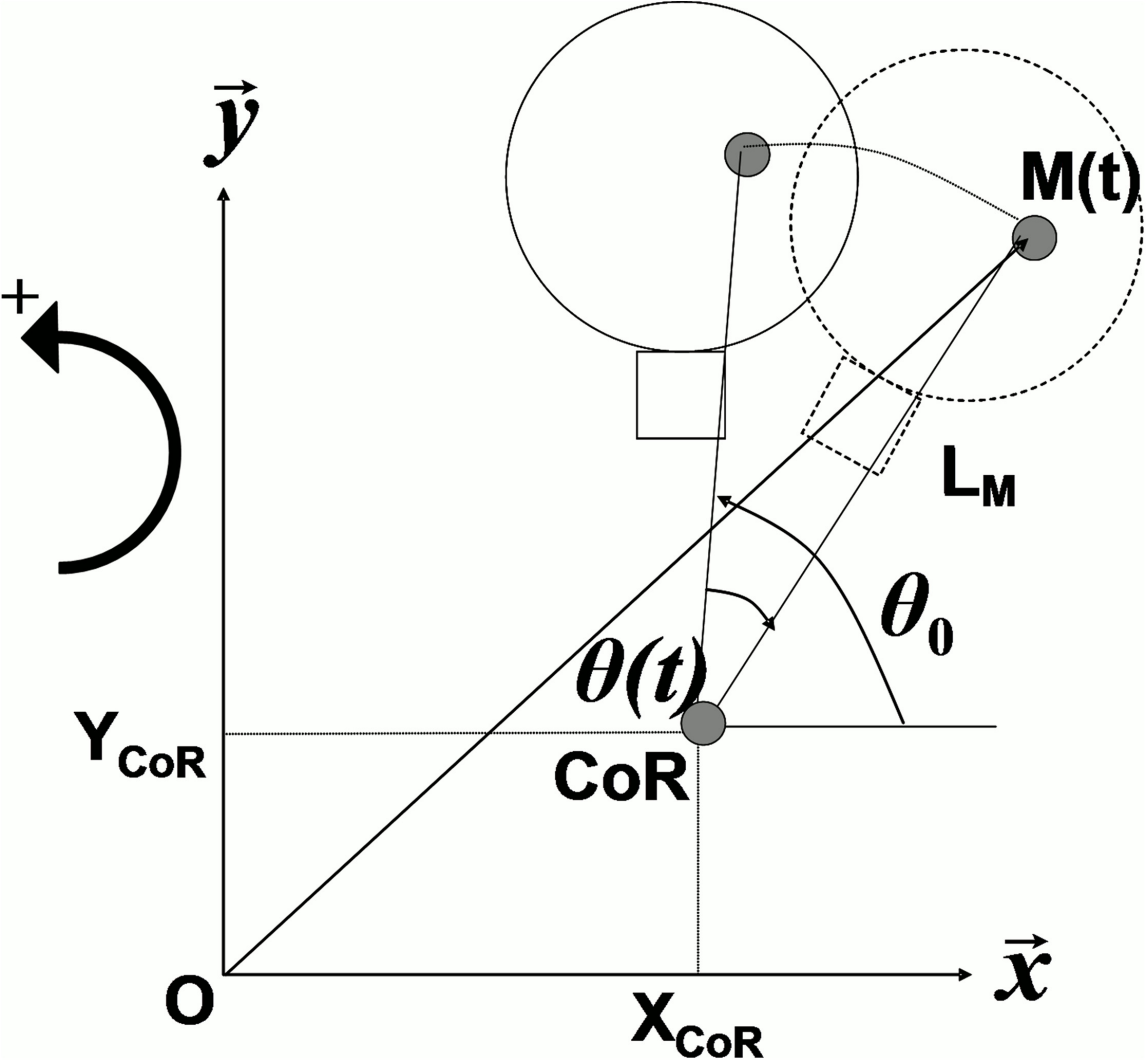
Configuration of head-neck segment rotation. M is a point of the head-neck rigid segment rotating around the resultant center of rotation (CoR) in $\left(\text{O},\vec{\text{X}},\vec{\text{Y}},\vec{\text{Z}}\right)$. Here, X_CoR_ and Y_CoR_ represent respectively horizontal and vertical CoR components, L_M_ is the radius of rotation of point M, θ_0_ is the initial angular position of point M, relative to the horizontal axis (X), and θ(t) is the angular position of point M, function of time (t), and relative to θ_0_. Anti-clockwise rotation is positive.

We used the methodology previously described by Portero et al. (2013) [9] to model the head-neck segment as a single joint system rotating around a resultant center of rotation (CoR). Considering the head-neck segment as a rigid body with one rotational degree of freedom, the position of any point of the body was expressed at each time as:

$$\vec{\text{OM}}(\text{t})=\left[{\text{X}}_{\text{CoR}}+{\text{L}}_{\text{M}}\;\text{cos}\left(\theta (\text{t})+\theta_{0}\right)\right]\vec{\text{x}}+\left[{\text{Y}}_{\text{CoR}}+{\text{L}}_{\text{M}}\;\text{sin}\left(\theta (\text{t})+\theta_{0}\right)\right]\vec{\text{y}}$$

where X_CoR_ and Y_CoR_ were respectively horizontal and vertical CoR components, L_M_ was the radius of rotation of a point M, θ_0_ was the initial angular position of point M, relative to the horizontal axis (X), and θ(t) was the angular position of point M, function of time (t), and relative to θ_0_, as described in Fig 2. The calculation of CoR during QR movement was formulated as an optimization problem with an objective function that evaluated the differences between experimental LEDs displacement measurements (with respect to shoulder position O) and theoretical positions of the corresponding points on rigid head-neck segment. It was mathematically formulated as:

Find optimal parameters ${\hat{\text{X}}}_{\text{CoR}},{\hat{\text{Y}}}_{\text{CoR}},{\hat{\text{L}}}_{\text{k}},{\hat{\theta }}_{0\text{k}},\hat{\theta }(\text{t})$ that minimize:

$$\text{C}=\frac{1}{2}\;\sum_{\text{t}}\sum_{\text{k}=1}^{{\text{N}}_{\text{LED}}}{\left[{\text{X}}_{\text{LEDk}}(\text{t})-\left({\hat{\text{X}}}_{\text{CoR}}+{\hat{\text{L}}}_{\text{k}}\;\text{cos}\left(\hat{\theta }(\text{t})+{\hat{\theta }}_{0\text{k}}\right)\right)\right]}^{2}+{\left[{\text{Y}}_{\text{LEDk}}(\text{t})-\left({\hat{\text{Y}}}_{\text{CoR}}+{\hat{\text{L}}}_{\text{k}}\;\text{sin}\left(\hat{\theta }(\text{t})+{\hat{\theta }}_{0\text{k}}\right)\right)\right]}^{2}$$

where X_LEDk_ and Y_LEDk_ were respectively horizontal and vertical kth LEDs measured positions N_LED_ = 5, ${\hat{\text{X}}}_{\text{CoR}}$ and ${\hat{\text{Y}}}_{\text{CoR}}$ were respectively optimal horizontal and vertical CoR components, ${\hat{\text{L}}}_{\text{k}}$ was the optimal radius of rotation of kth LED, ${\hat{\theta }}_{0\text{k}}$ was the optimal initial angular position of kth LED, and $\hat{\theta }(\text{t})$ was the optimal angular displacement of head-neck segment with $\hat{\theta }(0)=0$.

The isometric cervical flexion torque (T) developed by the head-neck segment rotating around the CoR was calculated as the product of F with the estimated lever arm, which is the vertical projection of the segment relating LED1 and the CoR (${\hat{\text{L}}}_{1}$) according to ${\hat{\theta }}_{01}$ defined as the estimated angle between the radius CoR—LED1 and the X axis:

$$\text{T}=\text{F}{\hat{\text{L}}}_{1}\;\text{sin}\;{\hat{\theta }}_{01}.$$

The inertia of the head-neck segment rotating around the CoR in the sagittal plane (I_CoR_) was calculated at the beginning of the QR movement when acceleration was maximal ${\ddot{\theta }}_{\text{max}}=\hat{\ddot{\theta }}(0)$, calculated from the second derivative of $\hat{\theta }(\text{t})$ obtained from the minimization of C, and static torque equals dynamic torque, using the following formula [8]:

$${\text{I}}_{\text{CoR}}=\frac{\text{T}}{{\ddot{\theta }}_{\text{max}}}.$$

In order to avoid damping, musculo-tendinous stiffness (S) was evaluated during the first 30 ms following the first acceleration peak after the release [30] as the change in torque versus change in angle by considering the formula [8]:

$$\text{S}=\frac{\Delta \text{T}}{\Delta \theta }=\frac{\Delta \ddot{\theta }\times {\text{I}}_{\text{CoR}}}{\Delta \theta }\;\text{where}\;\Delta \theta =\hat{\theta }(\text{t}=30\text{ms}),\;\text{and}\;\Delta \ddot{\theta }=\hat{\ddot{\theta }}(\text{t}=30\text{ms})-\hat{\ddot{\theta }}(\text{t}=0).$$

### Muscle EMG data

**Fig 3:**
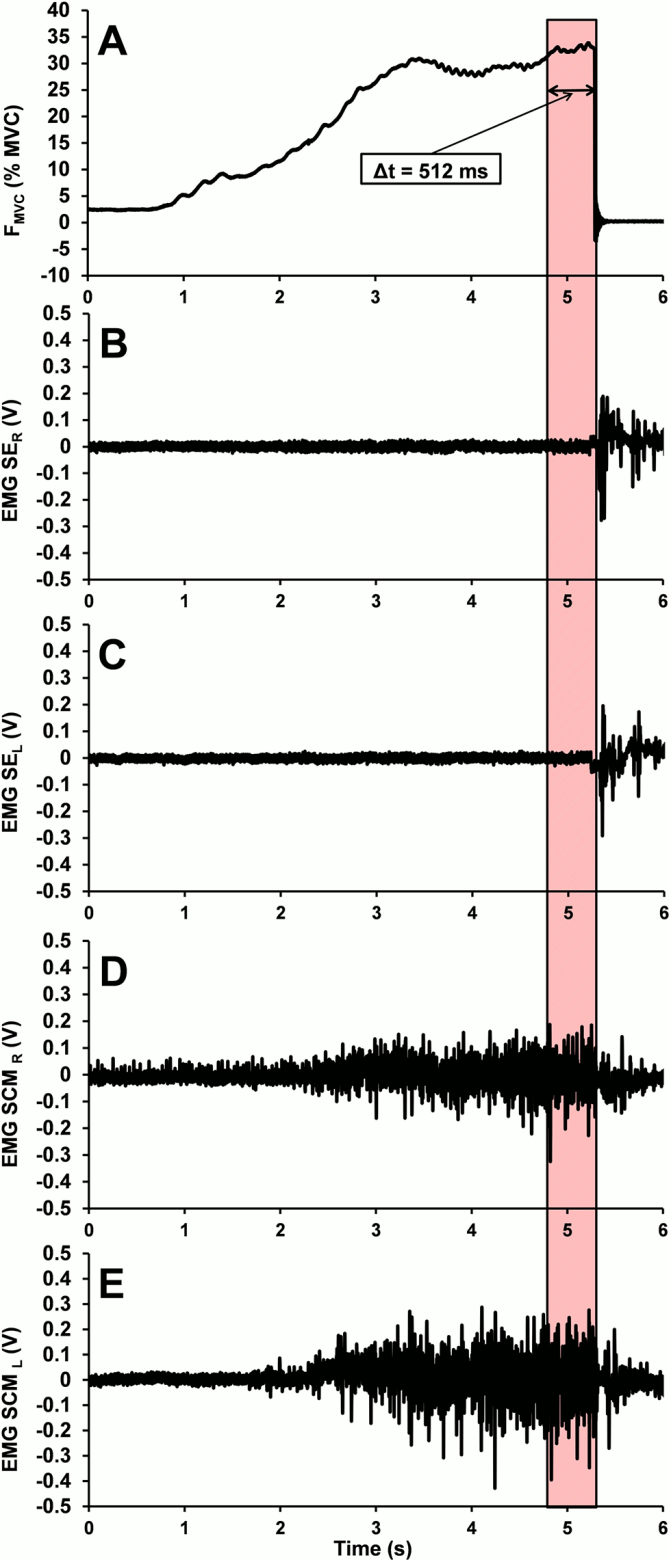
Force and EMG activity of cervical muscles. Typical normalized force according to MVC (F_MVC_) (A) and EMG recording on right cervical extensor (SE_R_), left cervical extensor (SE_L_), right cervical flexor (SCM_R_), and left cervical flexor (SCM_L_) (respectively B, C, D, and E) for one subject. EMG parameters were recorded over the last 512 ms of the isometric contraction (colored area) before the release during QR trial (here, 30% MVC trial).

For each test, the root mean square (RMS) and median power frequency (MDF) of the filtered EMG signal were computed. RMS was calculated within a 0.2 s sliding window and MDF was calculated on the power spectrum obtained using Welch’s periodogram method. Both were computed from EMG signals for the last 512 ms of the isometric contraction preceding the release (Fig 3). The RMS ratio between left antagonist muscles (SCM_L_ and ES_L_) and between right antagonist muscles (SCM_R_ and ES_R_) were estimated before and after the fatigue procedure.

### Statistical analysis

Means and standard deviations (SD) were calculated before and after the fatigue procedure across all the tests for S values and EMG parameters.

The normality assumption of the calculated data was checked with the Shapiro-Wilk test for paired data.

After that, the comparison of the values before and after the fatigue procedure was carried out using a paired *t* test for gaussian values and a Wilcoxon matched-pairs signed-ranks test for non-gaussian values. Values of the *t* test are noted t and values of the Wilcoxon test are noted z.

The slopes of the linear regression between S and T, for all QR trials of all subjects combined, were established as a stiffness index [31] before and after the fatigue procedure and compared with a paired *t* test.

All statistical analysis were performed at a significance level of P = 0.05.

## Results

### Musculo-tendinous stiffness

Values for S ranged from 46 Nm.rad^-1^ to 199 Nm.rad^-1^ (average = 111.15 ± 40.68 Nm.rad^-1^) before the fatigue procedure and from 30 Nm.rad^-1^ to 282 Nm.rad^-1^ (average = 113.69 ± 54.20 Nm.rad^-1^) after the fatigue procedure. These values were not significantly different after the fatigue procedure (t = 0.22; P = 0.83).

**Fig 4:**
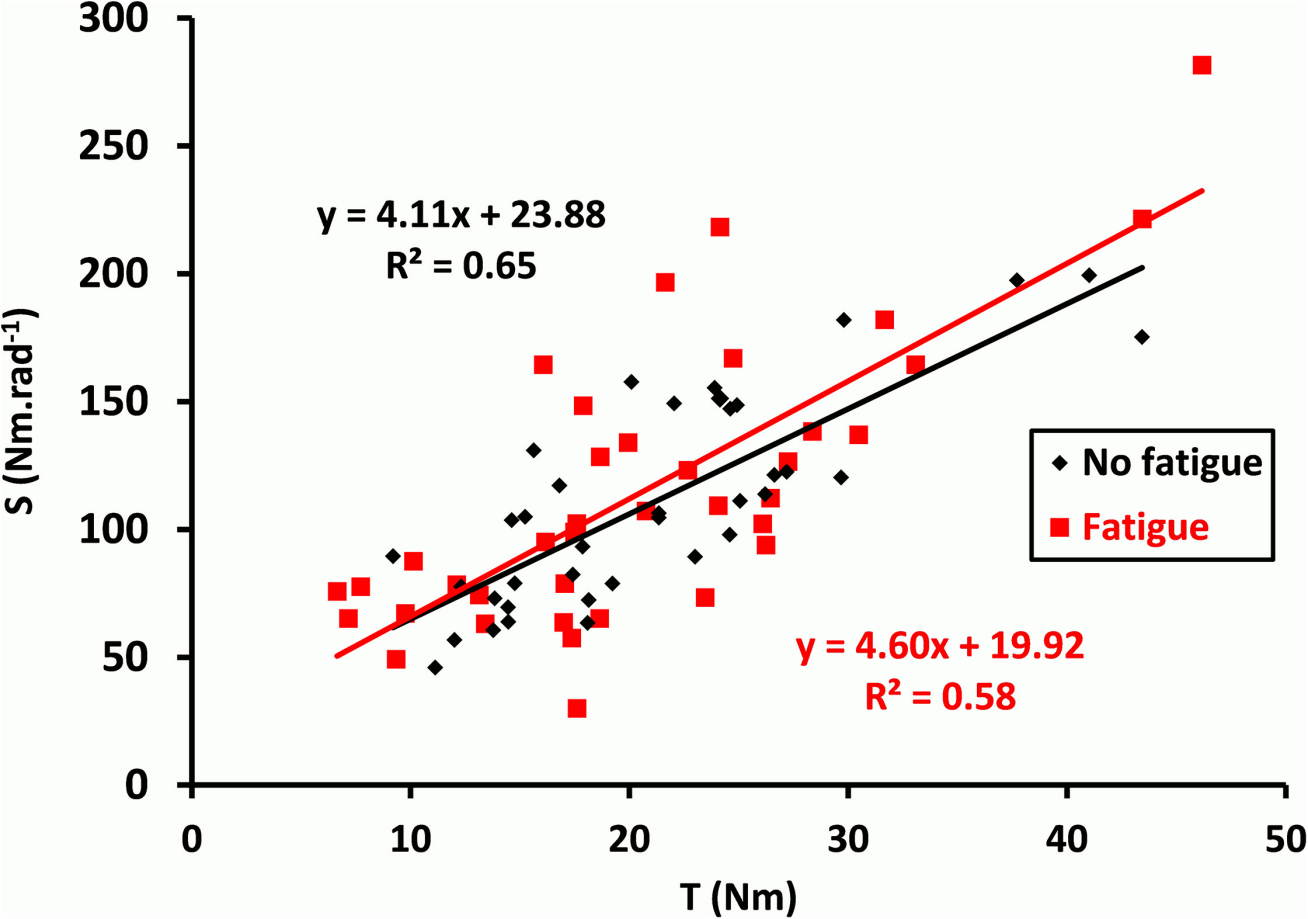
Evolution of stiffness index with fatigue. The relationship between the head-neck segment musculo-tendinous stiffness (S) and the isometric cervical flexion torque developed before the QR (T) for all subjects before and after the fatigue procedure (No fatigue and Fatigue, respectively). Linear regression showed a significant and linear increase of S according to T in the two conditions (P < 0.0001). There was no significant difference for the slopes of the linear regressions, providing the stiffness index of the head-neck segment in flexion, between the two conditions.

Fig 4 shows the linear regression between S and T before and after the fatigue procedure. It can be seen that there was a positive association between the SEC stiffness and the muscle torque for the two conditions (R^2^ = 0.65 and 0.58, respectively; P < 0.0001). The slope between S and T amounted to 4.11 rad^-1^ before the fatigue procedure, and to 4.60 rad^-1^ after the fatigue procedure. No significant differences for the stiffness index were noted with fatigue (t = 0.76; P = 0.45).

### Cervical muscles EMG

Mean and SD values of RMS for SE_L_, SE_R_, SCM_L_, and SCM_R_ before and after the fatigue procedure are reported in Table 1.

**Table 1:** Mean and SD values of EMG parameters of all muscles evaluated before and after the fatigue procedure (*P < 0.05).

	SE_L_		SE_R_		SCM_L_		SCM_R_	
	Before	After	Before	After	Before	After	Before	After
**RMS (mV)**	0.013 ± 0.004*	0.026 ± 0.024*	0.014 ± 0.005*	0.026 ± 0.018*	0.18 ± 0.11*	0.29 ± 0.17*	0.17 ± 0.10*	0.25 ± 0.13*
**MDF (Hz)**	57.92 ± 21.32	51.88 ± 19.00	51.91 ± 17.72	53.77 ± 20.62	82.89 ± 19.08*	72.44 ± 23.46*	82.32 ± 21.66*	69.28 ± 21.98*

**Fig 5:**
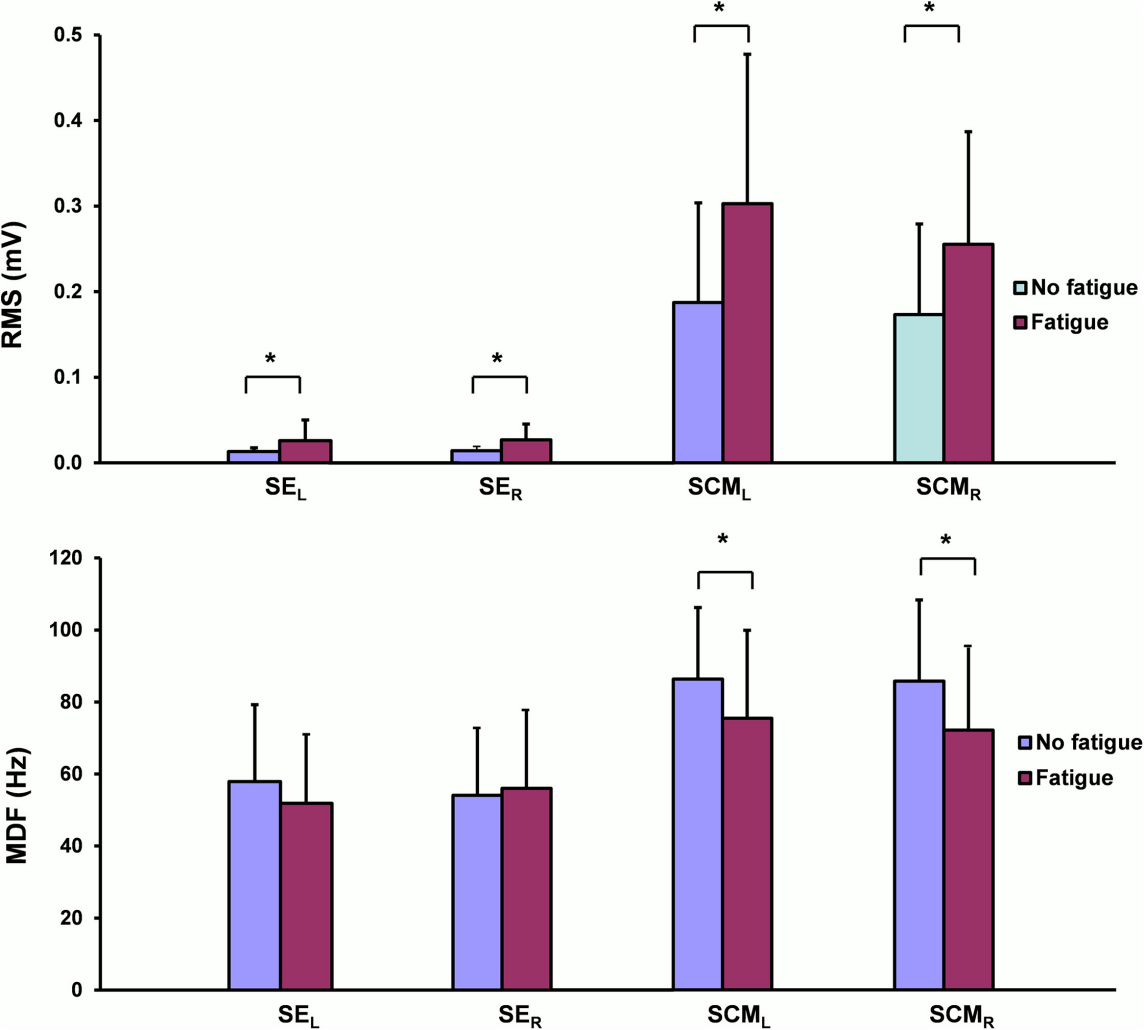
EMG parameters of cervical muscles. Evolution of RMS (top) and MDF (bottom) between before (No fatigue) and after (Fatigue) the fatigue procedure during the isometric contractions of QR (*P < 0.05).

A significant increase of the RMS was noted after the fatigue procedure on SE_L_ (z = -4.02; P < 0.0001), SE_R_ (z = -4.18; P < 0.0001), SCM_L_ (z = -3.92; P < 0.0001), and SCM_R_ (z = -3.70; P = 0.0002) (Fig 5).

The average RMS ratio of the left muscles was 15.21 ± 11.94 before the fatigue procedure and 15.05 ± 11.38 after the fatigue procedure. The average RMS ratio on the right muscles was 12.68 ± 5.62 before the fatigue procedure and 10.80 ± 4.62 after the fatigue procedure. No significant changes were noted for the ratio of RMS between SCM and SE for the left side (z = -0.98; P = 0.33) nor for the right side (z = -1.08; P = 0.28) after the fatigue procedure.

Mean and SD values of MDF for SE_L_, SE_R_, SCM_L_, and SCM_R_ before and after the fatigue procedure are reported in Table 1. A significant decrease was noted for MDF after the fatigue procedure for the SCM_L_ (z = -3.42; P = 0.0006) and for the SCM_R_ (z = -3.59; P = 0.0003). No significant changes were observed for MDF after the fatigue procedure for the SE_L_ (z = -0.88; P = 0.38) nor for the SE_R_ (z = -1.77; P = 0.08) (Fig 5).

## Discussion

Musculo-tendinous stiffness of cervical muscles is very important for head stabilization. Thus, the aim of this study was to estimate the effects of fatigue of cervical muscles on the head-neck segment musculo-tendinous stiffness. The musculo-tendinous stiffness was estimated using the procedure from Portero et al. (2013) [9], coupled with QR on the head-neck segment in flexion. Tests were performed before and after the execution of an adapted fatigue exercise protocol [29].

Results obtained on EMG data confirmed the effectiveness of the fatigue procedure. Fatigue of the SCM is demonstrated by concomitant significant increases of RMS values and decreases of MDF values on both sides. No fatigue was observed in the antagonist muscles, the SE. Conversely, we noticed a concomitant increase in the EMG amplitude of the SE antagonist muscle when fatigue developed in the SCM agonist muscles. This behavior has been observed by previous studies on trunk muscles where authors found modified muscle recruitment and increased coactivation to maintain spinal stability [23–25]. Our results confirmed a similar outcome for the head-neck segment. The analysis of the musculo-tendinous stiffness shows that its value increases with an increase in the exertion level. This result is congruent with other previous studies based on application of QR on different joint systems [30,31]. Current results displayed similar values of stiffness as previous ones showing the reliability of the method [9]. Moreover, no statistical differences were observed for changes on the stiffness index, neither on averaged stiffness values, nor on RMS ratio for fatigued muscles. This leads to the conclusion that the balance between musculo-tendinous stiffness of agonist muscles which generate the movement and antagonist muscles, which restrain the movement, is maintained at a specific level with cervical flexor fatigue. This tends to prove that, despite increased muscle impairment with fatigue, muscle changes are adapted accordingly in order to maintain the musculo-tendinous stiffness at a constant level.

We propose, therefore, that the highest priority of the central nervous system, by adapting the ratio between agonist and antagonist muscles, is to stabilize the head early by reaching a constant stiffness value regardless of muscle impairment. This result is novel and seems to be unique to the head-neck segment. Indeed, literature shows contrasted results concerning stiffness changes with fatigue, but no study displayed results with a constant stiffness value with fatigue. Several authors [17] note an increase in stiffness in isolated rat muscle, whereas others observed a decrease for the elbow extensors [13,14]. However, we should remain cautious when comparing our results with these studies since important discrepancies in experimental procedures may be noted (e.g. oscillation perturbations versus QR, sub-maximal versus maximal force level, isolated animal muscle versus *in situ* human evaluation).

Ultimately, based on our results, we can propose that an adapted stabilization pattern exists for the head-neck segment considering its importance for proprioception, sensory data integration, and protection from spinal cord injuries. This pattern is based on modulation of muscle activity to produce a constant stiffness value. In addition, we could hypothesize that this modulation of agonist activity may facilitate the induction of their reflex activity to protect the cervical spine under fatigue conditions.

We have performed EMG measurement of only two pairs of the most accessible cervical muscles involved in head stabilization [32]. It is not unlikely that other muscles may be involved in the task and may modulate results. Therefore, confirmation with further studies should be performed by recording different cervical muscles to better characterize the muscle involvement with fatigue.

In conclusion, this is the first study investigating the relationship between fatigue and musculo-tendinous stiffness on cervical muscles. It shows that stiffness values and stiffness indexes remain unchanged with fatigue. In addition, cervical flexor fatigue increases the participation of antagonist muscles. It can be concluded that the motor adaptation to counterbalance the effects of fatigue is to adapt the net involvement of agonist and antagonist muscles in order to maintain the segment stiffness parameters at a constant level. This result suggests that the stiffness of the head-neck segment may reflect an active process associated with the modulation of the agonist and antagonist muscles involvement to stabilize the head-neck segment. The stiffness seems to be a strong invariant parameter, regardless of fatigue conditions. For the motor system, it could be of greatest importance to keep the stiffness parameter unchanged in order to optimize the head segment stabilization. This result has never been published before and it is of great importance in order to better understand head stabilization. Further studies should be performed to confirm this proposition, for example with fatigue localized in the extensors.
